# Heritable Transmission of Stress Resistance by High Dietary Glucose in *Caenorhabditis elegans*

**DOI:** 10.1371/journal.pgen.1004346

**Published:** 2014-05-01

**Authors:** Arnaud Tauffenberger, J. Alex Parker

**Affiliations:** 1 CRCHUM, Montréal, Québec, Canada; 2 Département de pathologie et biologie cellulaire, Montréal, Québec, Canada; 3 Département de neurosciences, Université de Montréal, Montréal, Québec, Canada; University of Cambridge, United Kingdom

## Abstract

Glucose is a major energy source and is a key regulator of metabolism but excessive dietary glucose is linked to several disorders including type 2 diabetes, obesity and cardiac dysfunction. Dietary intake greatly influences organismal survival but whether the effects of nutritional status are transmitted to the offspring is an unresolved question. Here we show that exposing *Caenorhabditis elegans* to high glucose concentrations in the parental generation leads to opposing negative effects on fecundity, while having protective effects against cellular stress in the descendent progeny. The transgenerational inheritance of glucose-mediated phenotypes is dependent on the insulin/IGF-like signalling pathway and components of the histone H3 lysine 4 trimethylase complex are essential for transmission of inherited phenotypes. Thus dietary over-consumption phenotypes are heritable with profound effects on the health and survival of descendants.

## Author Summary

Nutritional state has major effects on health and longevity, and investigations into the mechanisms of dietary restriction have taken the lion's share of recent genetic discoveries. We used *Caenorhabditis elegans* to investigate the role of diet on nematode physiology and report the surprising finding that exposure to high glucose at one generational time point has heritable effects in descendent progeny. Glucose promotes resistance against cellular stress and neurodegeneration in parental and descendent progeny, while reducing lifespan only in the parental generation. Furthermore, we found that glucose mediated protection is dependent on well-known metabolic and stress response genes. Numerous strategies have evolved to ensure reproductive success in the face of changing and challenging environments. It is believed that extended lifespan phenotypes observed under dietary restriction conditions maximize an organism's survival until environmental conditions improve allowing for reproduction. We discovered a novel diet-influenced reproductive advantage; animals subjected to high dietary glucose are resistant to protein damaging stress, and this resistance is transmitted to their progeny. The trade-off for stress-resistant progeny is decreased lifespan and fecundity in the parental strain suggesting that this strategy may be adaptive under nutrient rich conditions.

## Introduction

Aging is an inevitable process that affects all organisms and a better understanding of the underlying biological mechanisms is relevant to human health [1]. In nature, organisms struggle against environmental conditions to survive and hopefully reproduce. This is an energetically costly and persistent process, thus nutrient availability greatly influences an organism's life history with profound affects on survival, reproduction and lifespan. The life history of most organisms naturally consists of periods of low nutrient availability and core mechanisms have evolved to deal with nutrient stress, namely starvation or near-starvation. Research into the genetic underpinnings of nutritional state on health and longevity is an active area of research, with the mechanisms of dietary restriction taking the lion's share of recent genetic discoveries [2]. Modern industrialized societies no longer live in fear of famine, but instead live in conditions of a near perpetual feast. Unfortunately, diets high in sugar are linked to numerous health problems in humans [3]. However, we wondered why animal species will over-consume resources if given the opportunity and hypothesized there may be an adaptive benefit to such behavior.

Using *Caenorhabditis elegans* to investigate over-consumption phenotypes we discovered that exposure to high glucose concentrations at one generational time point, the parental generation, had persistent and heritable effects in descendent progeny. Glucose promotes resistance against cellular stress and neurodegeneration in parental and descendent progeny, while reducing lifespan in the parental generation only. Furthermore, we found that glucose mediated phenotypes are dependent on known metabolic genes including components of the Insulin/IGF-like pathway, the sirtuin *sir-2.1*, and AMPK, while the transgenerational inheritance of glucose-directed phenotypes are dependent on histone methylation enzymes. Thus, dietary glucose can induce the transmission of heritable, cellular phenotypes with profound consequences on health and survival.

## Results

### Heritable diminution of progeny from glucose exposure in the parental generation

**Figure 1:**
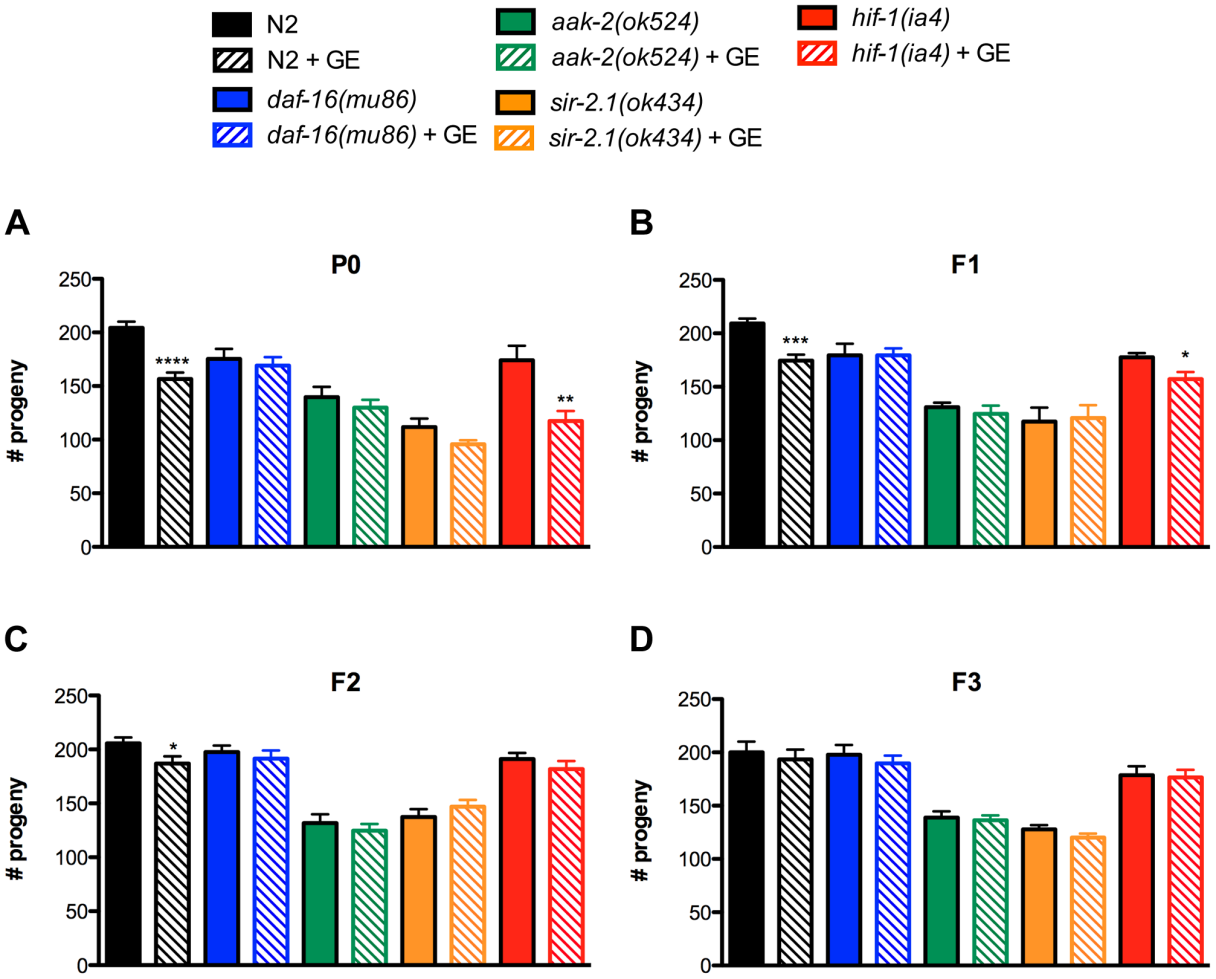
Heritable diminution of progeny from glucose exposure in the parental generation. (A) In parental P0 generation animals, glucose enrichment (GE) decreased the average number of progeny of wild type N2 and *hif-1* mutant worms compared to untreated controls. GE had no effect on *daf-16*, *aak-2* or *sir-2.1* mutants. **P<0.01 versus untreated *hif-1* controls, ****P<0.0001 versus untreated N2 controls (B) F1 generation N2 and *hif-1* descendants had reduced progeny numbers compared to F1 descendants from untreated P0 controls. *P<0.05, ***P<0.001 (C) N2 worms in the F2 generation from P0 parents exposed to GE also had reduced progeny numbers. *P<0.05 (D) F3 generation descendants from GE treated P0 parents had comparable progeny numbers compared to animals descendent from untreated P0 parents.

We first focused on reproduction since in *C. elegans* the average number of progeny is a strong physiological phenotype showing transgenerational inheritance [4], and wild type N2 worms cultured under glucose enrichment (GE) conditions have reduced progeny numbers [5]–[7]. Consistently we observed that parental generation (P0) N2 worms exposed to GE had reduced total progeny numbers compared to untreated controls [5] (Figure 1A), and that this effect extended to the descendent F1 and F2 generations (Figure 1B–D). Thus, glucose can induce a heritable, transgenerational phenotype on progeny from a single exposure of the P0 generation.

The insulin/IGF-signalling pathway is an evolutionarily conserved network of genes regulating an organism's response to nutritional states and is a major conserved regulator of aging [2], thus we investigated its contribution to the transgenerational effects of GE on progeny numbers. DAF-16 is a forkhead transcription factor and the major downstream regulator of the insulin/IGF-like signalling pathway [8], [9] and we observed that GE did not further reduce progeny in *daf-16* mutants at any generational time point (Figure 1). We then examined genes known to respond to nutritional status and interact with both *daf-16* and the insulin/IGF-like signalling pathway including *aak-2*, which encodes the alpha subunit of the AMP-activated protein kinase (AMPK) [10], and *sir-2.1* that encodes an orthologue of the histone deacetylase SIRT1 [11]. Similar to *daf-16* mutants, we observed that mutation in either *aak-2* or *sir-2.1* likewise blocked the progeny reducing effects of GE. Finally, we examined *hif-1*, the *C. elegans* orthologue of mammalian hypoxia induced factor 1 (HIF1) [12], a protein that regulates glucose metabolism and the cellular response to low oxygen conditions and observed that GE continued to reduce progeny numbers in the P0 and F1 generation of *hif-1* mutants.

Exposure to high dietary glucose reduces the lifespan of wild type N2 worms, as well as the long-lived phenotype of worms with hypomorphic mutations in the gene encoding the worm's sole Insulin/IGF-like receptor DAF-2 [5]–[7]. We confirmed that GE reduced the lifespan of N2 and *daf-2* worms in the P0 generation but found no evidence that this was a transgenerational phenotype since the F1 and F2 descendent progeny had lifespans similar to untreated control worms (Figure S1, Table S1). In total, these data suggest that specific components of the insulin/IGF-like signalling pathway regulate the negative effects of GE on reproduction in *C. elegans* and that the heritable effects on reproduction can be separated from lifespan.

### Transgenerational inheritance of resistance to oxidative stress

**Figure 2:**
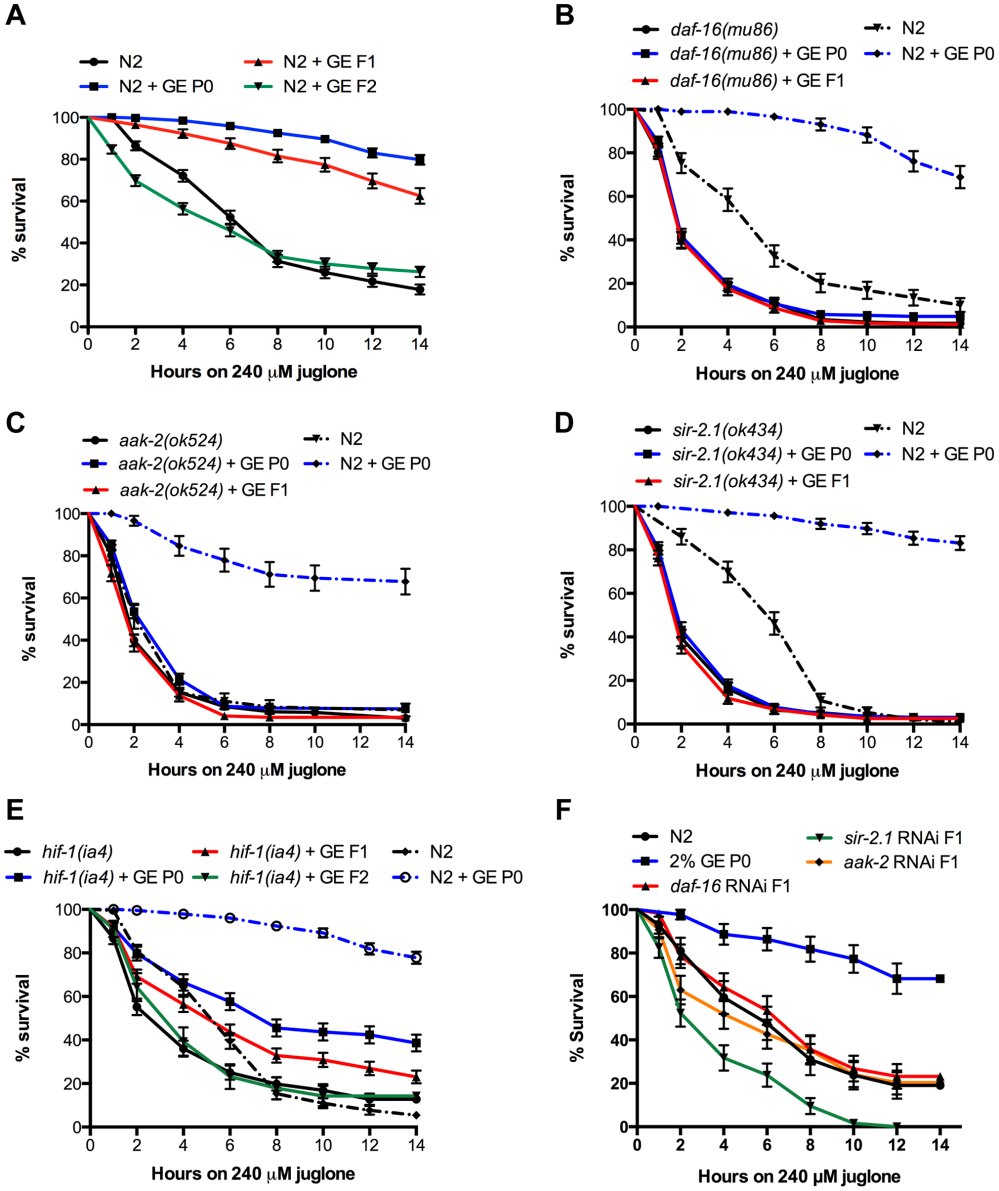
Transgenerational inheritance of resistance to oxidative stress. (A) N2 animals exposed to GE are highly resistant to juglone-induced lethality and this resistance was transmitted to descendent progeny in the F1 and generation, P<0.0001 versus untreated animals. (B–E) Resistance to oxidative stress by GE was lost in the P0 and F1 generations in animals mutant for (B) *daf-16*, (C) *aak-2*, or (D) *sir-2.1*. (E) GE continued to provide resistance to P0 and F1 animals mutant for *hif-1*, P<0.0001 versus untreated animals. (F) GE increased oxidative stress resistance in P0 N2 animals but this effect was lost in F1 animals treated with *daf-16*, *aak-2* or *sir-2.1* RNAi clones.

Despite the negative effects on fecundity and lifespan, we previously reported that GE strongly protected worms against environmental stress [5]. We tested for resistance to oxidative stress using juglone, a natural product from the black walnut tree that produces intracellular oxidative stress and decreases the survival of N2 worms [13]. Treating P0 N2 worms with glucose provided potent protection against oxidative stress induced lethality, and this protection persisted into the F1 generation of N2 worms even though these F1 animals were never exposed to glucose (Figure 2A). Glucose-mediated resistance against oxidative stress was not transmitted further since the F2 generation of N2 worms was sensitive to juglone (Figure 2A). Having observed that a carbohydrate supplemented diet augmented stress resistance phenotypes, we wondered if this was due to a contribution from the worms' bacterial food source and/or and if other dietary supplements like protein or fat would also promote stress resistance. We discounted possible bacterial effects since GE continued to promote resistance to oxidative stress for worms grown on heat-killed bacteria (Figure S2A). Additionally, we observed no augmented resistance to oxidative stress for worms grown on plates supplemented with either methionine or oleic acid [14] (Figure S2B) suggesting the stress resistance phenotypes may be limited to dietary sugars.

We then tested if insulin/IGF-like pathway genes were required for protection against juglone by glucose and observed that similar to the effects on progeny numbers, *daf-16*, *aak-2* and *sir-2.1* were all required for the heritable protective effects of GE against oxidative stress (Figure 2B–D). Consistent with the effects of GE on progeny and lifespan, *hif-1* was not required for the glucose-mediated protection against glucose (Figure 2E). To further confirm that these genes were required for transmission, we treated F1 animals with RNAi against *daf-16, aak-2 and sir-2.1* and RNAi knockdown of each of these genes blocked the heritable transmission of stress resistance (Figure 2F). These data suggest that key components of the insulin/IGF-like pathway are required for the heritable, protective effects of glucose against oxidative stress. Recent work showed a possible hormetic protection in response to oxidative stress, as a mild induction of the cellular stress response could increase long term resistance and longevity [15]. To investigate the possible role of glucose as a stress inducer, we stained the worms with dihydrofluorescein, a marker of oxidative stress [16]. We observed comparable levels of fluorescence for N2 worms treated with glucose compared to untreated control worms (Figure S2C) suggesting that glucose does not induce a generalized oxidative stress phenotype.

### Parental exposure to glucose provides transgenerational protection against neurodegeneration

**Figure 3:**
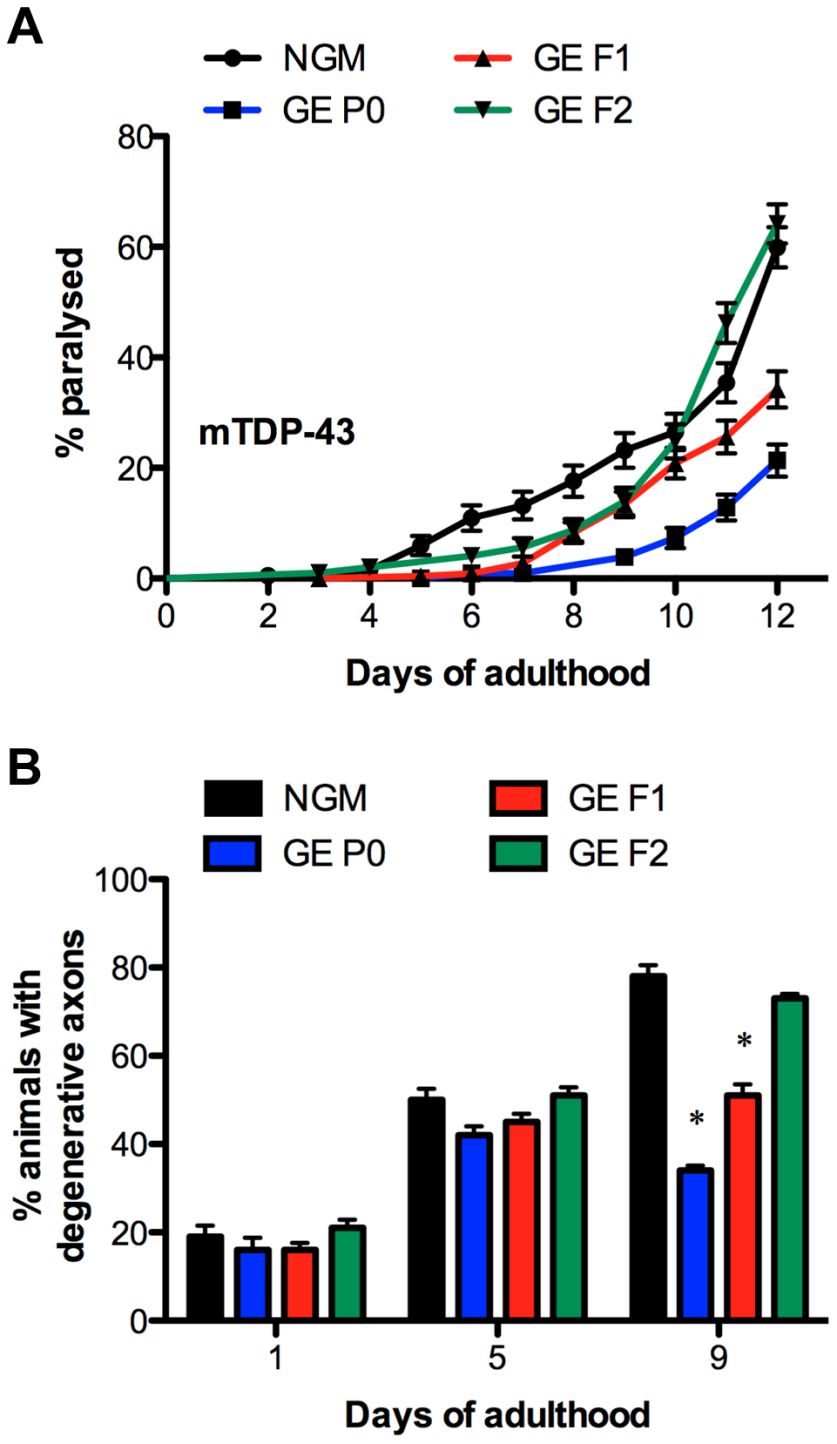
Parental exposure to glucose provides transgenerational protection against neurodegeneration. (A–B) GE reduces TDP-43 mediated age-dependent (A) paralysis, P<0.0001 versus untreated animals and (B) neurodegeneration in P0 animals and their F1 descendants, *P<0.0001 versus untreated animals.

Aging is a risk factor for many diseases including late-onset neurodegenerative disorders [17]. To determine whether or not glucose could provide generational protection against age-dependent proteotoxicity we turned to a well-characterized *C. elegans* model of TAR DNA-binding protein 43 (TDP-43) motor neuron toxicity [18]–[21]. TDP-43 is a conserved RNA/DNA binding protein with mutant variants being causative for amyotrophic lateral sclerosis [22]. *C. elegans* expressing mutant TDP-43 in motor neurons show adult onset, age-dependent paralysis and neurodegeneration phenotypes that are diminished by treatment with glucose [5]. We observed that P0 generation TDP-43 worms treated with glucose had reduced rates of paralysis (Figure 3A) and axonal degeneration (Figure 3B) compared to untreated controls, and this protective effect persisted into the F1 generation. Thus, GE can reduce genetically encoded proteotoxicity and this effect is heritable. We next confirmed that GE did not reduce the paralysis rate through a hormetic stress response as dihydrofluorescein levels were indistinguishable from mutant TDP-43 animals treated with glucose (Figure S2C), but also because glucose-mediated neuroprotection was not lost after treatment with the antioxidant N-acetyl cysteine (Figure S2D).

### Transmission of glucose phenotypes requires H3K4me3 components

**Figure 4:**
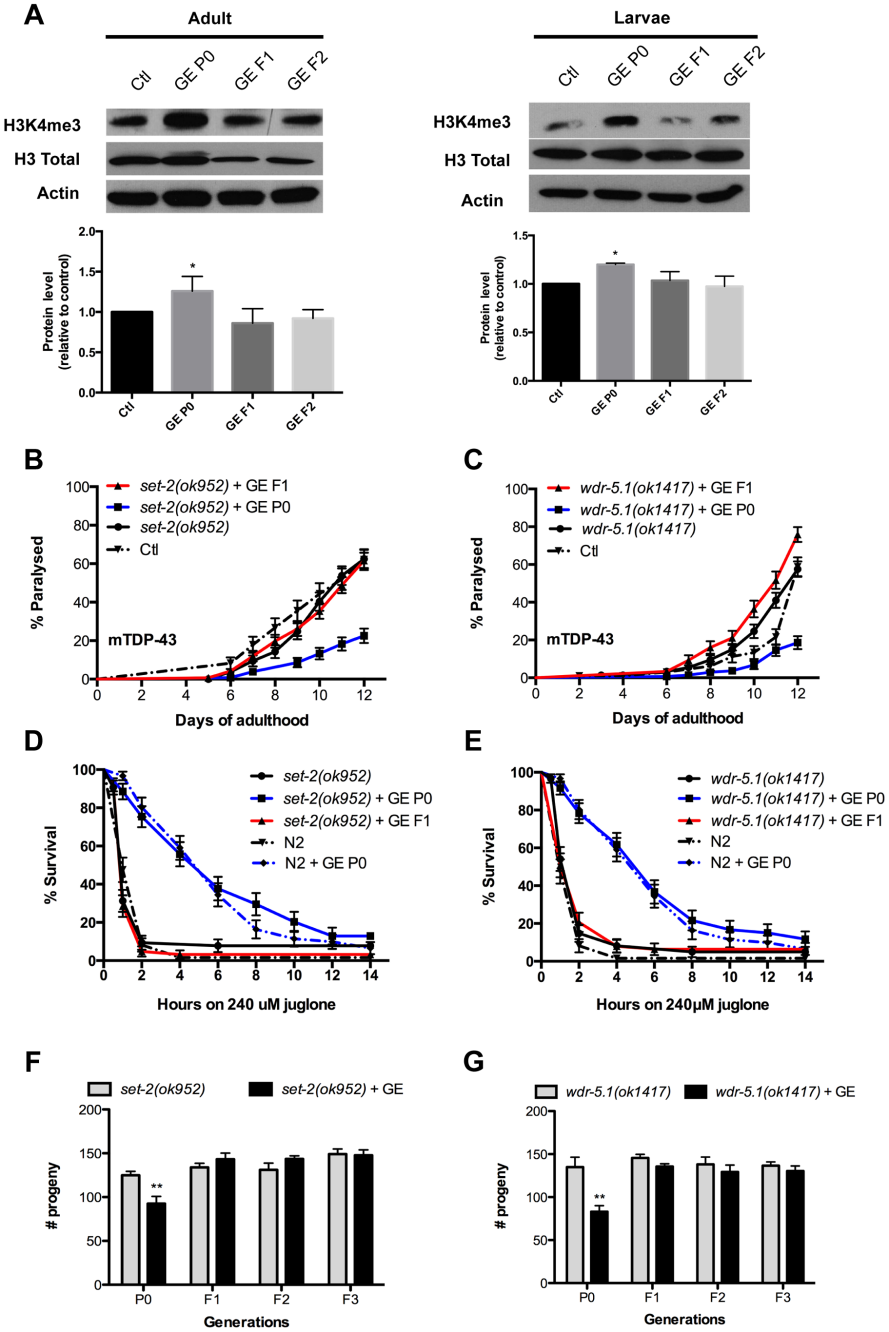
Transgenerational inheritance of glucose phenotypes requires H3K4me3 components. (A) N2 animals treated with 2% GE had an increased H3K4me3 mark and this effect was lost in subsequent generations. (B–C) GE delayed late onset paralysis in P0 but not in F1 generation of mTDP-43; *set-2(ok952)* (B) and mTDP-43; *wdr-5.1(ok1417)* (C) animals compared to untreated control and this protection was lost in F1 generation. (D–E) Stress resistance was increased in P0 but not in F1 generation of COMPASS (Complex Proteins Associated with Set1) mutants (D) *set-2(ok952)* and (E) *wdr-5.1(ok1417)*. (F–G) Total progeny numbers were reduced in P0 but not in F1 generations of (F) *set-2* and (G) *wdr-5.1* mutants.

Work from Brunet and colleagues demonstrated that components of the histone H3 lysine 4 trimethylation (H3K4me3) complex are essential to the transmission of transgenerational effects on longevity [23]. Thus, we hypothesized that genes encoding the H3K methyltransferase *set-2*, and the H3K4me3 complex component *wdr-5.1* would be required for the heritable transmission of glucose phenotypes on neurodegeneration, stress resistance and fecundity. First, we observed that H3K4me3 methylation was increased in P0 animals compared to untreated controls (Figure 4A). We observed this methylation mark in both young worms at the L3 larval stage and adults, thus suggesting that tissue heterogeneity or the presence of eggs in older animals do not contribute to this phenomenon. However, the increased H3K4me3 methylation observed in P0 animals was not transmitted to the F1 or F2 generations (Figure 4A). These observations are in agreement with work from Brunet and colleagues [23], where the H3K4me3 complex is associated with the transgenerational inheritance of longevity, but the H3K4me3 mark is not heritable. One interpretation is that H3K4me3 may be an indirect effect arising from other functions of the H3K4me3 complex in regulating global physiological changes.

We further investigated the genetic requirements of *set-2* and *wdr-5.1* for the transmission of glucose-mediated phenotypes. First we observed that GE suppressed the rate of paralysis in mutant TDP-43; *set-2* and mutant TDP-43; *wdr-5.1* mutants in the P0 generation, but suppression of mutant TDP-43-induced paralysis was not transmitted to the descendent F1 generation (Figure 4B and 4C). Accordingly, GE reduced axonal degeneration in P0 animals but failed to rescue the degeneration in F1 animals carrying *set-2(ok952)* or *wdr-5.1(ok1417)* mutations (Figure S3A). These data suggest that *set-2* and *wdr-5.1* are required for the heritable transmission of stress resistance phenotypes, but are not necessary for the stress resistance itself. To confirm this we tested *set-2* and *wdr-5.1* mutants, subjected to GE, against oxidative stress and observed that GE continued to provide protection against juglone in the P0 generation, but this protection was lost in the F1 generation (Figure 4D and 4E) and this phenomenon was confirmed by RNAi treatment against *set-2* and *wdr-5.1* (Figure S3B and S3C). Likewise with the negative effects on reproduction, *set-2* and *wdr-5.1* mutants treated with GE had reduced numbers of progeny in the P0 generation, but progeny numbers returned to normal levels in the F1 descendants and subsequent F2 and F3 generations (Figure 4F and 4G).

However, the requirement of *set-2* and *wdr-5.1* for the transmission of glucose phenotypes may be independent of H3K4me3 methylation status. We confirmed that *set-2* and *wdr-5.1* are indeed required for the increased H3K4me3 methylation induced by glucose of the P0 generation of animals, since this methylation mark was abolished by *set-2* or *wdr-5.1* mutations (Figure S3D). Despite this, we observed that H3K4me3 methylation was not transmitted from P0 to F1 animals (Figure 4A) suggesting that this mark is not associated with the heritable transmission of glucose-induced phenotypes. Thus *set-2* and *wdr-5.1* may have activities independent of H3K4me3 methylation for the heritable transmission of glucose-associated phenotypes.

### The germline is required for transmission of glucose protection

**Figure 5:**
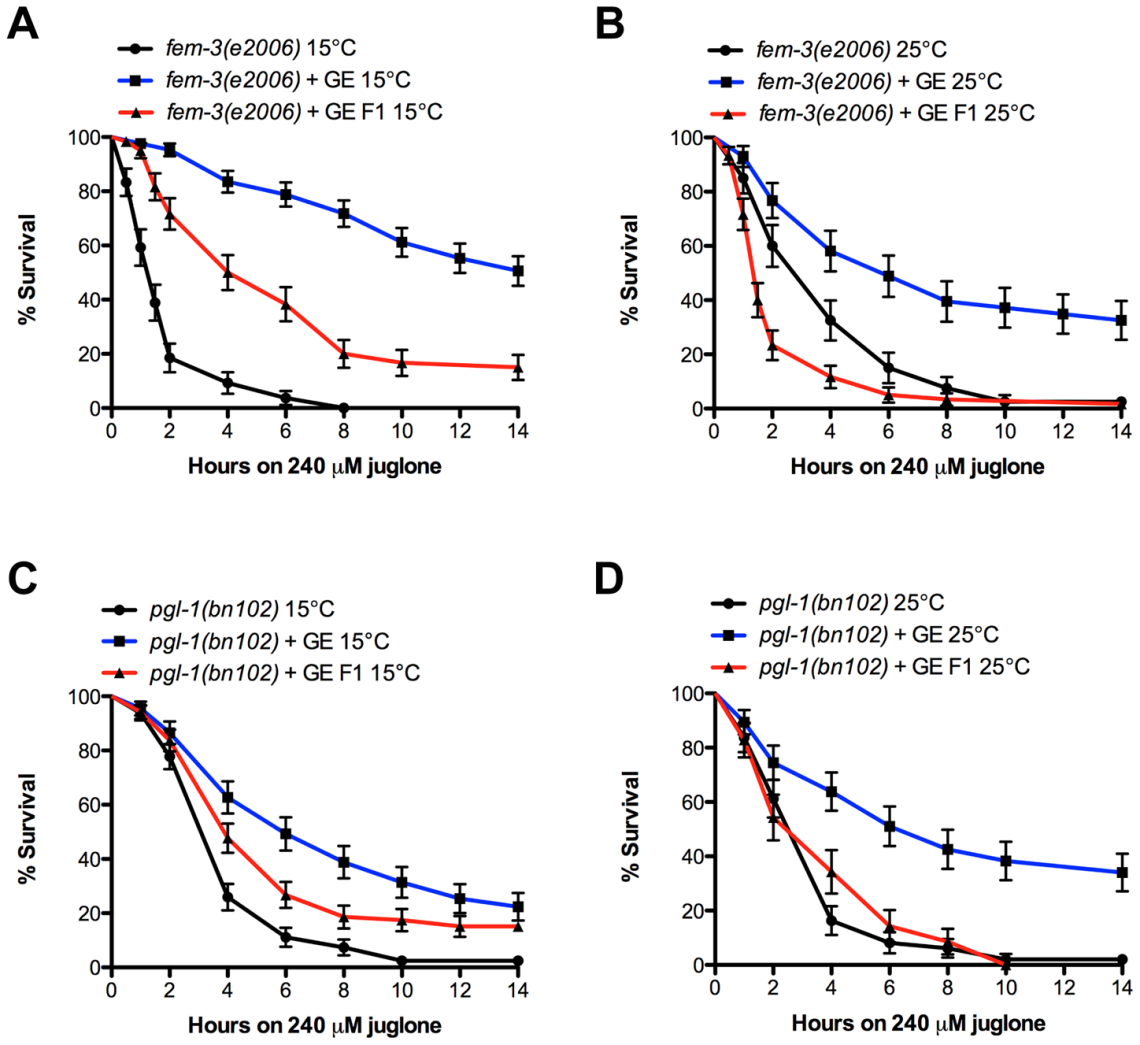
The germline is required for transmission of glucose protection. (A–B) Glucose increased stress resistance in P0 and F1 generation of *fem-3(e2006)* mutants at 15°C, but failed in the F1 generation at 25°C. (C–D) Glucose increased resistance to juglone in P0 and F1 generation of *pgl-1(bn102)* mutants at 15°C but failed in the F1 generation at 25°C.

The transgenerational inheritance of longevity requires a functional germline [23]. Thus, we investigated the role of the germline in transmission of stress resistance after GE by using feminized mutants *fem-3(e2006)* that are not able to produce mature eggs at restrictive temperatures [24] and *pgl-1(bn102)* that is not able to form a functional germline at restrictive temperatures [25]. Both strains showed increased resistance to juglone under GE conditions in the P0 and F1 generation at 15°C, but the transmission was lost at 25°C in the F1 generation, indicating that the glucose-induced transmission of stress resistance is dependent on a functional germline (Figure 5).

## Discussion

Numerous strategies have evolved to ensure reproductive success for organisms in the face of changing and challenging environments [1]. It is believed that extended lifespan phenotypes observed under dietary restriction conditions maximize an organism's survival until environmental conditions improve allowing for reproduction. We discovered a novel diet-influenced reproductive advantage; animals subjected to high dietary glucose are resistant to protein damaging stress, and this resistance is transmitted to their progeny. The trade-off for stress-resistant progeny is decreased lifespan and fecundity in the parental strain suggesting that this strategy may be adaptive under nutrient rich conditions.

In the P0 generation, high dietary glucose leads to negative effects on lifespan and reproduction along with increased protection against protein-damaging stress. The resulting F1 progeny are resistant to stress via an epigenetic mechanism along with a small reduction in fecundity, but these animals do not suffer negative lifespan effects. This dietary induced adaptation may be favorable from an evolutionary standpoint where in times of plenty an epigenetic program is initiated to maximize the survival of progeny at the expense of less total progeny and a decrease in parental health. This mechanism is reminiscent of antagonistic pleiotropy, where fitness in early life is increased at the expense of negative effects on health and fitness in late life [2]. Here, excess energy from high dietary glucose may be used to maintain proteostasis under stress conditions [5], but only the parental P0 hermaphrodites pay the price of shortened lifespan. The recently proposed theory of hyperfunction may help explain these contradictions [26], where the continued activity of developmental and growth programs later in life leads to decreased fitness and longevity [2]. Indeed, the majority of genes regulating lifespan have metabolic functions essential for development or growth, and perhaps the continued activity of these networks driven by excess dietary glucose leads to negative phenotypes. If true, we would expect that only those animals directly exposed to glucose would suffer negative effects and this is seen by the large reduction of lifespan in P0, but not F1 animals.

While the reduced progeny phenotype persists for two generations, the stress resistance phenotypes last only until the F1 generation, suggesting maternal inheritance rather than a transgenerational inheritance mechanism. Furthermore, we observe increased H3K4me3 methylation only in the parental P0 animals exposed to glucose, but not in the descendent F1 and F2 progeny. Thus, while the stress resistance phenotypes are passed to the F1 generation, the glucose induced methylation mark is not, so perhaps maternally deposited RNAs contribute to the glucose-induced phenotypes of the F1 progeny. One interpretation might be that H3K4me3 occurs on specific loci, and thus is impossible to detect by western blot. Alternatively, the complex may initiate large-scale physiological changes, and H3K4me3 may be an indirect, non-heritable effect simply indicating open chromatin and increased transcription. Finally, it is possible that an additional methylation mark, like H3K36me3 is involved [27] in the transmission of glucose-associated phenotypes. In any event, both histone modifying enzymes and the insulin/IGF-like pathway are necessary for the transmission of glucose-induced phenotypes. This is likely an adaptive phenotype since while the dietary status of the P0 animals may be predictive for the immediate environment of F1 animals, this may not be the case for F2 generations and beyond.

Of interest is the comparison of phenotypes of high dietary glucose conditions versus nutrient stress from reduced feeding. Worms experiencing dietary restriction are long-lived [28], have reduced fertility [29], show resistance to thermal stress [30] but are not resistant to genetically encoded neuronal proteotoxicity [5]. These observations suggest that the bulk of dietary-restricted animals' resources are used to maintain survival, minimizing reproductive potential until environmental conditions improve. In contrast, high dietary glucose may represent a nutrient rich environment, and these animals have the luxury to enlist genetic programs promoting survival of their progeny.

Given that an organism's life history is greatly affected by nutritional status, an open question is how far do cellular changes go in response to nutritional state. The emerging field of transgenerational epigenetic inheritance is providing evidence that certain epigenetic modifications persist over several generations [31]. Furthermore, the investigation of heritable phenotypic effects from sustained dietary changes is a developing field that may have implications on human health [32]–[34]. The investigation of the biological consequences of early environmental influences has a long history [35], and there are numerous studies into the effects of prenatal diet on the health of offspring, including mammals [36], but the molecular mechanisms are not well known. Future studies in genetically tractable models like *C. elegans* will be a powerful approach to unravel the epigenetic mechanisms of nutrient stress on healthy aging.

## Materials and Methods

### Worm strains and genetics

Standard methods of culturing and handling worms were used. Worms were maintained on standard NGM plates streaked with OP50 *E. coli.* In some experiments D-glucose was added to NGM plates (all products from Sigma-Aldrich). All strains were scored at 20°C unless indicated. Mutations and transgenes used in this study were: *daf-16(mu86), sir-2.1(ok434), aak-2(ok524), hif-1(ia4), set-2(ok952), wdr-5.1(ok1417), fem-3(e2006), pgl-1(bn102) and xqIs133[unc-47::TDP-43[A315T];unc-119(+)]*. Some strains were provided by the *C. elegans* Genetics Center (University of Minnesota, Minneapolis), which is funded by NIH Office of Research Infrastructure Programs (P40 OD010440). Mutants or transgenic worms were verified by visible phenotypes, PCR analysis for deletion mutants, sequencing for point mutations or a combination thereof. Deletion mutants were out-crossed a minimum of three times to wild type worms prior to use.

### Worm behavioral tests

Mutant TDP-43 animals were scored for paralysis and counted as positive if they failed to move upon prodding with a worm pick. Worms were scored as dead if they failed to move their head after being prodded on the nose and showed no pharyngeal pumping. For the paralysis tests worms were grown on NGM or NGM +2% glucose and transferred to NGM-FUDR or NGM-FUDR +2% glucose. For the F1 generation, L4 animals were transferred from NGM +2% glucose to NGM and their progeny used as the F1 generation. The same method was used for the F2 generation.

### Fluorescence microscopy

For scoring of neuronal processes from mTDP-43 transgenics, animals were selected at days 1, 5 and 9 of adulthood for visualization of motor neurons processes *in vivo*. Animals were immobilized in M9 with 5 *mM* levamisole and mounted on slides with 2% agarose pads. Neurons were visualized with a Leica 6000 and a Leica DFC 480 camera. A minimum of 100 animals was scored per treatment over 4–6 trials. The mean and SEM were calculated for each trial and two-tailed t-tests were used for statistical analysis.

### Stress assays

For oxidative stress tests, worms were grown on NGM or NGM with a dietary supplement (glucose, oleic acid or methionine) and transferred to NGM plates +240 µM juglone at adult day 1. For the F1, L4 worms from NGM +2% glucose plates were transferred on NGM and their progeny used as F1 generation. The same process was used for the F2 generation. Worms were evaluated for survival every 30 min for the first 2 hours and every 2 hours after up to 14 hours. Nematodes were scored as dead if they were unable to move in response to heat or tactile stimuli. For all tests worms, 20 animals/plate by triplicates were scored. Temperature sensitive mutant *fem-3(e2006)* or worms were maintained at 15°C and switched to 25°C at hatching and kept at this temperature until tested on juglone. Temperature sensitive *pgl-1(bn102)* were maintained at 15°C and switched to 25°C at the L4 larvae stage and kept at this temperature until tested on juglone.

### Lifespan assays

Worms were grown on NGM or NGM +4% glucose and transferred on NGM-FUDR or NGM-FUDR + glucose. For the F1 generation, L4 animals from the NGM +4% glucose plates were transferred to NGM plates and progeny used as the F1 generation on NGM-FUDR. The same process was used to prepare the F2 generation. 20 animals/plate by triplicates were tested at 20°C from adult day 1 until death. Worms were scored as dead if they didn't respond to tactile or heat stimulus.

### Progeny tests

For scoring progeny, 10 L4 worms were grown on NGM or NGM +2% glucose and placed at 20°C. Over the next three days individual worms were transferred to new plates and the L1 larvae were scored for each plate. For the F1 generation, 10 L4 larvae from the P0 were transferred to new NGM plates without glucose and this process was repeated for the F2 and F3 generations.

### Dihydrofluorescein diacetate assay

For visualization of oxidative damage in the transgenic strains the worms were incubated on a slide for 30 minutes with 5 µM dihydrofluorescein diacetate dye (Sigma-Aldrich) and then washed with 1× PBS three times. After the slide was fixed fluorescence was observed with the Leica system described above.

### Worm lysates

Worms were collected in M9 buffer, washed 3 times with M9 and pellets were placed at −80°C overnight. Pellets were lysed in RIPA buffer (150 mM NaCl, 50 mM Tris pH 7.4, 1% Triton X-100, 0.1% SDS, 1% sodium deoxycholate)+0.1% protease inhibitors (10 mg/ml leupeptin, 10 mg/ml pepstatin A, 10 mg/ml chymostatin LPC;1/1000). Pellets were passed through a 27_1/2_ G syringe 10 times, sonicated and centrifuged at 16000 *g*. Supernatants were collected.

### Protein quantification

All supernatants were quantified with the BCA Protein Assay Kit (Thermo Scientific) following the manufacturer's instructions.

### Immunoblot

Worm RIPA samples (50 µg/well) were resuspended directly in 1× Laemmli sample buffer, migrated in 14% polyacrylamide gels, transferred to nitrocellulose membranes (BioRad) and immunoblotted. Antibodies used: rabbit anti-Histone H3 Total (1∶1000, ab1791 Abcam), rabbit anti-Histone tri-methylated (1∶1000, ab8580 Abcam), and mouse anti-actin (1∶5000 , MP Biomedicals). Blots were visualized with peroxidase-conjugated secondary antibodies and ECL Western Blotting Substrate (Thermo Scientific). Densitometry was performed with Photoshop (Adobe).

### RNAi experiments

RNAi-treated strains were fed with *E. coli* (HT115) containing an Empty Vector (EV), *set-2* (C26E6.9), *wdr-5.1* (C14B1.4), *daf-16* (R13H8.1) or *aak-2* (T01C8.1) RNAi clones from the ORFeome RNAi library and *sir-2.1* (R11A8.4) clone from the Ahringer RNAi library. RNAi experiments were performed at 20°C. Worms were grown on either NGM or NGM +2% glucose both enriched with 1 mM Isopropyl-b-D-thiogalactopyranoside (IPTG).

### Statistics

For paralysis and stress-resistance tests, survival curves were generated and compared using the Log-rank (Mantel-Cox) test, and 60–100 animals were tested per genotype and repeated at least three times.

## Supporting Information

Figure S1 Lifespan reduction by glucose is not transmitted over generation. (A–B) GE reduced lifespan in the P0 generation of (A) N2 and (B) *daf-2(e1370)* animals, P<0.0001, but failed to reduce lifespan in the F1 and F2 generations. (Related to Figure 1).
(TIF)

Figure S2 Glucose protection is independent of a hormetic increase of oxidative stress. (A) N2 animals exposed to GE on dead OP50 bacteria (DB) were highly resistant to juglone-induced lethality and this resistance was transmitted to descendent progeny in the F1 and generation, P<0.0001 versus untreated animals. (B) Methionine (0.1%) and oleic acid (0.45 mM) failed to increase resistance to juglone. (C) Images of adult worms stained with 5 µM dihydrofluorescein diacetate. N2 worms do not show increased fluorescence after treatment with glucose. mTDP-43 animals experience high levels of oxidative stress and strongly fluoresce when stained with dihydrofluorescein diacetate. (D) GE reduced the paralysis rate of mTDP-43 animals and treatment with N-acetyl cysteine did not block the suppression of paralysis.
(TIF)

Figure S3 COMPASS genes are required for methylation increase by glucose. (A) Glucose failed to increase the methylation H3K4me3 mark in *set-2(ok952)* and *wdr-5.1(ok1417)* mutated animals (Related to Figure 4). (B) Glucose enrichment reduced axonal degeneration in mTDP-43; *set-2(ok952)* (P<0.001) and mTDP-43; *wdr-5.1(ok1417)* (P<0.0001) P0 animals but failed to rescue the phenotype in the F1 generation. (C–D) RNAi against (C) *set-2* and (D) *wdr-5* failed to block GE protection against juglone in P0 animals, but blocked the transmission in the F1 generation. (Related to Figure 4D and 4E). The N2 control was used for both experiments.
(TIF)

Table S1 Lifespan analysis for all experiments. Related to Figure S1. Animals that died prematurely (ruptured, internal hatching) or were lost (crawled off the plate) were censored at the time of scoring. All control and experimental animals were scored and transferred to new plates at the same time. n.s. not significant.
(PDF)
