# Supplementary figures and images for: Heritable Transmission of Stress Resistance by High Dietary Glucose in Caenorhabditis elegans
Source: PLoS Genet. 2014 May 1;10(5):e1004346. doi: 10.1371/journal.pgen.1004346 (PMC4006733; doi:10.1371/journal.pgen.1004346)

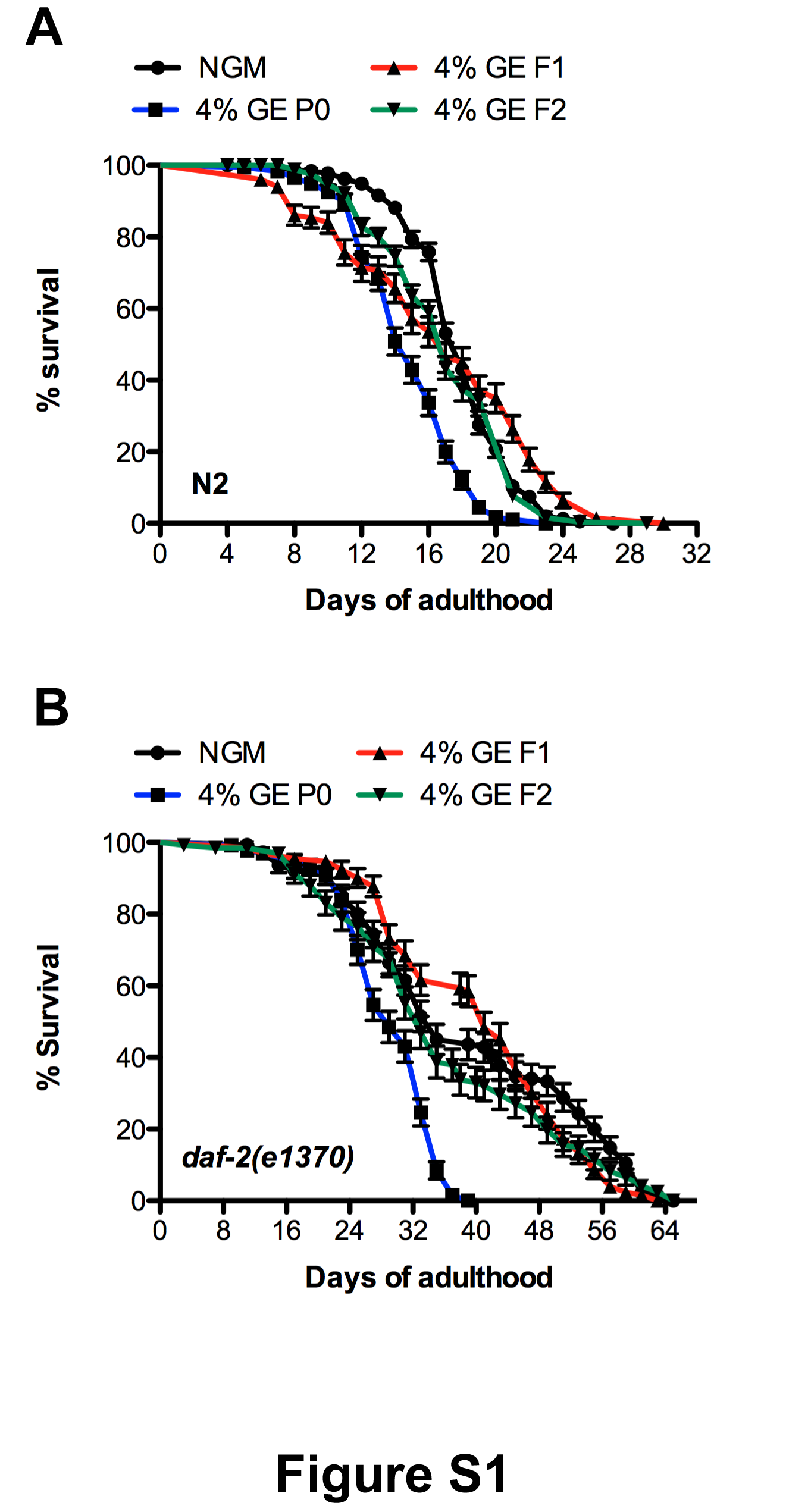

Supplement: Figure S1 — Lifespan reduction by glucose is not transmitted over generation. (A–B) GE reduced lifespan in the P0 generation of (A) N2 and (B) daf-2(e1370) animals, P<0.0001, but failed to reduce lifespan in the F1 and F2 generations. (Related to Figure 1). (TIF) [file pgen.1004346.s001.tif]

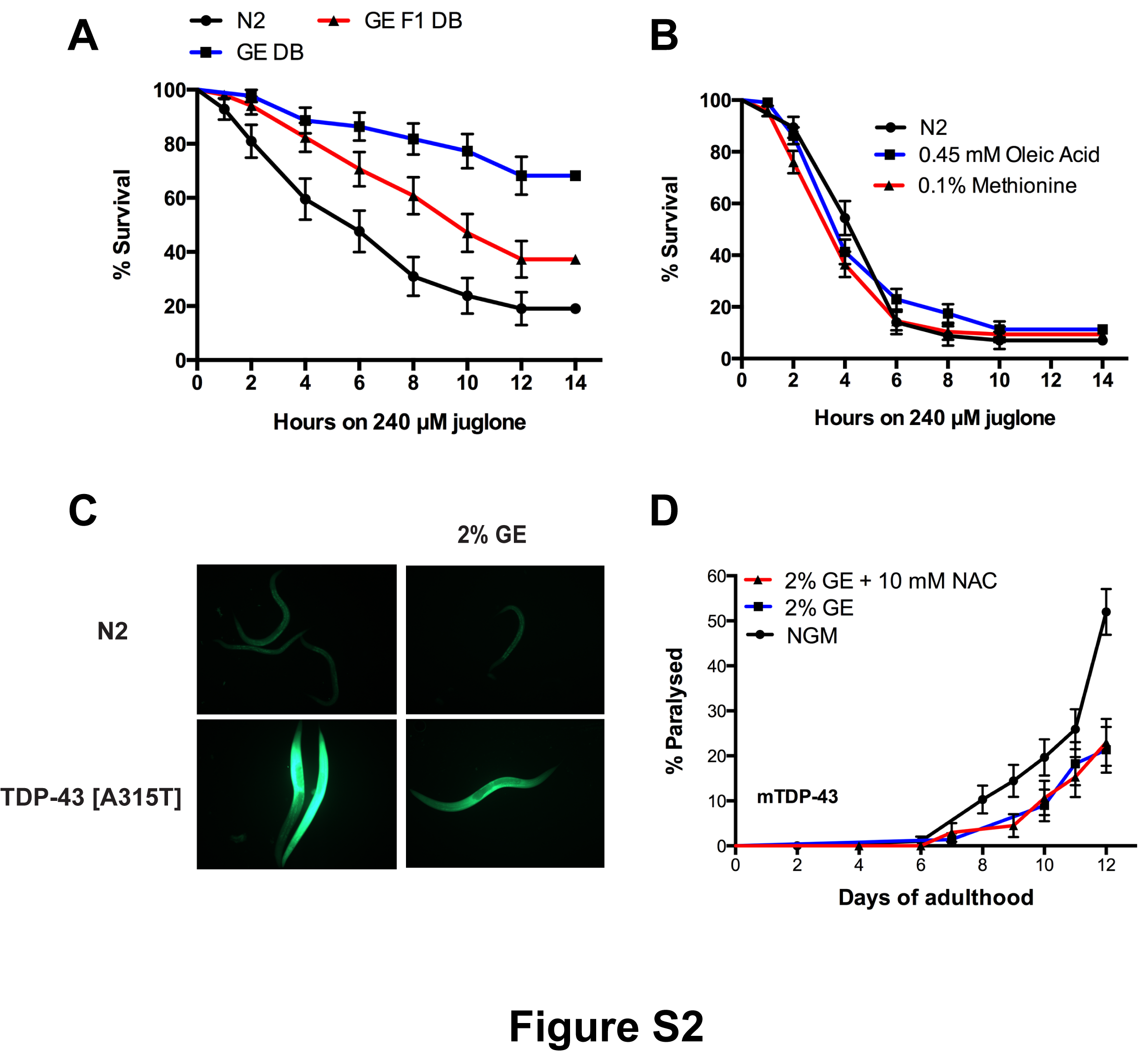

Supplement: Figure S2 — Glucose protection is independent of a hormetic increase of oxidative stress. (A) N2 animals exposed to GE on dead OP50 bacteria (DB) were highly resistant to juglone-induced lethality and this resistance was transmitted to descendent progeny in the F1 and generation, P<0.0001 versus untreated animals. (B) Methionine (0.1%) and oleic acid (0.45 mM) failed to increase resistance to juglone. (C) Images of adult worms stained with 5 µM dihydrofluorescein diacetate. N2 worms do not show increased fluorescence after treatment with glucose. mTDP-43 animals experience high levels of oxidative stress and strongly fluoresce when stained with dihydrofluorescein diacetate. (D) GE reduced the paralysis rate of mTDP-43 animals and treatment with N-acetyl cysteine did not block the suppression of paralysis. (TIF) [file pgen.1004346.s002.tif]

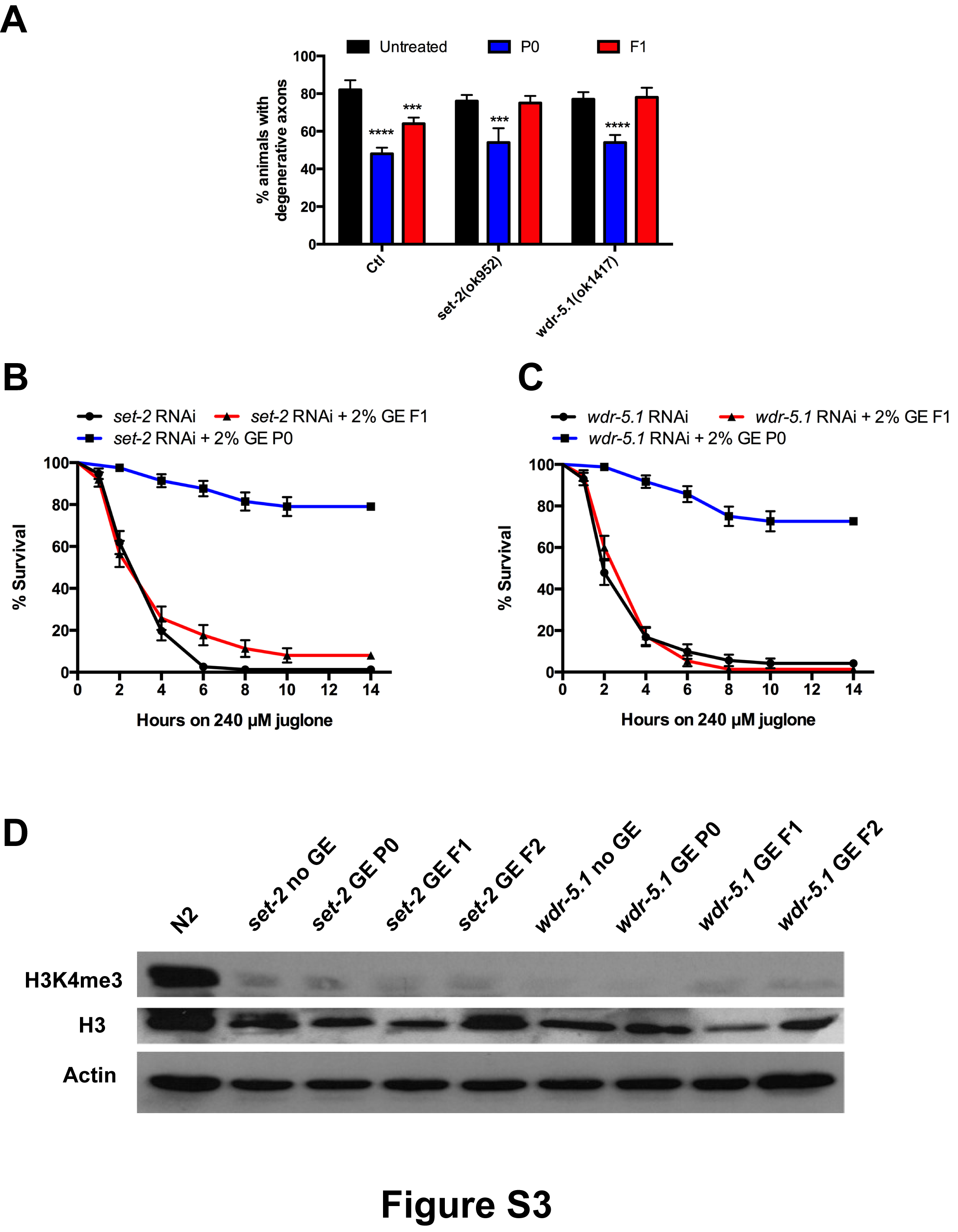

Supplement: Figure S3 — COMPASS genes are required for methylation increase by glucose. (A) Glucose failed to increase the methylation H3K4me3 mark in set-2(ok952) and wdr-5.1(ok1417) mutated animals (Related to Figure 4). (B) Glucose enrichment reduced axonal degeneration in mTDP-43; set-2(ok952) (P<0.001) and mTDP-43; wdr-5.1(ok1417) (P<0.0001) P0 animals but failed to rescue the phenotype in the F1 generation. (C–D) RNAi against (C) set-2 and (D) wdr-5 failed to block GE protection against juglone in P0 animals, but blocked the transmission in the F1 generation. (Related to Figure 4D and 4E). The N2 control was used for both experiments. (TIF) [file pgen.1004346.s003.tif]
