# Supplementary material for: Heritable Transmission of Stress Resistance by High Dietary Glucose in Caenorhabditis elegans
Source: PLoS Genet. 2014 May 1;10(5):e1004346. doi: 10.1371/journal.pgen.1004346 (PMC4006733; doi:10.1371/journal.pgen.1004346)
Supplement: Table S1 — Lifespan analysis for all experiments. Related to Figure S1. Animals that died prematurely (ruptured, internal hatching) or were lost (crawled off the plate) were censored at the time of scoring. All control and experimental animals were scored and transferred to new plates at the same time. n.s. not significant. (PDF) [file pgen.1004346.s004.pdf]

Strain and treatment	Mean lifespan	P value	75th percentile (days)	Maximum lifespan	Total number of deaths/total
N2	18		18	27	309/317
N2 + 4% GE P0	15	**<0,0001**	16	21	175/175
N2 + 4% GE F1	17	n.s. 0,1083	19	29	142/152
N2 + 4% GE F2	17	n.s. 0,2327	18	30	244/257
daf-2(e1370)	35		53	65	138/142
daf-2(e1370) + GE P0	29	**<0,0001**	33	39	130/132
daf-2(e1370) + GE F1	41	n.s. 0,2965	49	63	128/132
daf-2(e1370) + GE F2	33	n.s. 0,1353	47	65	122/129

Table S1
